# Therapeutic Plasma Exchange and Multiple Sclerosis Dysregulations: Focus on the Removal of Pathogenic Circulatory Factors and Altering Nerve Growth Factor and Sphingosine-1-Phosphate Plasma Levels

**DOI:** 10.3390/cimb45100489

**Published:** 2023-09-25

**Authors:** Dimitar Tonev, Albena Momchilova

**Affiliations:** 1 Department of Anesthesiology and Intensive Care, University Hospital “Tzaritza Yoanna—ISUL”, Medical University of Sofia, 1527 Sofia, Bulgaria; 2 Institute of Biophysics and Biomedical Engineering, Bulgarian Academy of Science, 1113 Sofia, Bulgaria; albena_momchilova@abv.bg

**Keywords:** multiple sclerosis, immune pathophysiology, autoantibodies, nerve growth factor, sphingosine-1-phosphate, therapeutic plasma exchange, neuroprotection

## Abstract

Multiple sclerosis (MS) is predominantly an immune-mediated disease of the central nervous system (CNS) of unknown etiology with a possible genetic predisposition and effect of certain environmental factors. It is generally accepted that the disease begins with an autoimmune inflammatory reaction targeting oligodendrocytes followed by a rapid depletion of their regenerative capacity with subsequent permanent neurodegenerative changes and disability. Recent research highlights the central role of B lymphocytes and the corresponding IgG and IgM autoantibodies in newly forming MS lesions. Thus, their removal along with the modulation of certain bioactive molecules to improve neuroprotection using therapeutic plasma exchange (TPE) becomes of utmost importance. Recently, it has been proposed to determine the levels and precise effects of both beneficial and harmful components in the serum of MS patients undergoing TPE to serve as markers for appropriate TPE protocols. In this review we discuss some relevant examples, focusing on the removal of pathogenic circulating factors and altering the plasma levels of nerve growth factor and sphingosine-1-phosphate by TPE. Altered plasma levels of the reviewed molecular compounds in response to TPE reflect a successful reduction of the pro-inflammatory burden at the expense of an increase in anti-inflammatory potential in the circulatory and CNS compartments.

## 1. Introduction

Multiple sclerosis (MS) is an autoimmune multifocal central nervous system (CNS) inflammatory disease, characterized by chronic inflammation, demyelination, axonal damage, and subsequent gliosis. The disease is of unknown etiology with a possible genetic predisposition and effect of certain environmental factors [1,2]. Although there is no complete consensus regarding the pathological processes leading to MS, it is generally accepted that the disease begins with an autoimmune inflammatory reaction targeting oligodendrocytes (OLs) followed by a rapid depletion of OLs regenerative capacity with subsequent permanent neurodegenerative changes and disability [3]. Therefore, the therapeutic efforts should be focused with priority on the first stage of demyelination, when the damage is not yet irreversible. The most efficient treatment modality for neurodegeneration remains an early and aggressive anti-inflammatory intervention, because the prevention of tissue injury may best control the escalating T-cell-driven and bystander B-cell activation, ongoing breakdown of blood–brain barrier (BBB) and epitope spreading, that may perpetuate neuroaxonal damage [4]. After the impressive efficacy of anti-CD20 antibody therapy for patients with a relapsing–remitting form of the disease, there is renewed attention to B cells in the pathogenesis of MS [5]. Recent research highlights the central role of B lymphocytes in the development of MS lesions, in particular the main role of IgG and IgM in newly forming lesions [6]. Thus, early and aggressive control of antibodies contributing to oligodendrocyte and axonal damage in MS becomes of utmost importance. However, the questionable efficacy of anti-CD20 therapy in reducing the antibody levels [7], along with its delayed onset of action compared to the rapid action of therapeutic apheresis, raised issues of combination therapies in general [8] and between apheresis and anti-CD20 antibody therapy in particular [9]. On the other hand, although pathogenetically justified, the role of apheresis (or therapeutic plasma exchange (TPE), a term used interchangeably) in MS patients still has a limited and even questioned application [7,10]. As usually happens, the latter critical review could pave the way for searching for new answers. It was suggested that further studies should be undertaken to determine the levels and precise effects of both beneficial and harmful components in sera of MS patients during different phases of the disease in terms of their capability to serve as markers for appropriate TPE protocols [10]. The aim of our review is to discuss some relevant examples of the proposed field of new research, focusing on the removal of pathogenic circulating factors and altering the plasma levels of nerve growth factor (NGF) and sphingosine-1-phosphate (S1P) by TPE and their impact on MS dysregulations.

In this focus review, after the short summary of the pathogenesis, we explore the relevant data on autoantibodies, NGF, S1P, and TPE regarding MS, from both detrimental and beneficial points of view.

## 2. Methodology

A literature search was conducted through June 2023 of MEDLINE, EMBASE, and Cochrane Library, based on Medical Subject Heading (MeSH) of “therapeutic plasma exchange”, “nanomembrane-based”, “plasmapheresis”, “apheresis”, immuno-mediated”, “autoimmune”, “neurological”, “disorders”, “diseases”, “Multiple Sclerosis”, “MS”, “acute”, “chronic”, “relapsing remitting”, “secondary progressive”, “primary progressive”, “aggressive”, “attacks”, “exacerbations”, “relapses”, “Central Nervous System”, “CNS”, “dysregulations”, “inflammation”, “neuro-inflammation”, “degeneration”, “neuro-degeneration”, “demyelination”, “myelination”, “remyelination”, “neuroprotection”, “immunomodulation”, “oxidative stress”, “axons”, “neurons”, “conductivity”, “T cell”, “B cells”, “activation”, “glia”, “microglia”, “oligodendrocytes”, “OLs”, “oligodendrocyte progenitor cells”, “OPCs”, “astrocytes”, “polarization”, “cytokines”, “chemokines”, “pro-inflammatory”, “anti-inflammatory”, “autoantibodies”, “pathogenic”, “antigens”, “epitopes”, “complement”, “immune complexes”, “Nerve Growth Factor”, “NGF”, “neurotrophins”, “receptors”, “tropomyosin receptor kinase A”, “TrkA”, “p75 neurotrophin receptor”, “p75NTR”, “Sphingosine-1-Phosphate”, “S1P”, “S1P receptor”, “S1PR”, “S1PR1”, “S1PR2”, “S1PR3”, “S1PR4”, “S1PR5”, “plasma levels”, as well as by manual search in the local database. The search had no language restrictions.

## 3. Pathogenesis of MS

### 3.1. Between Space and Time Axes

**Figure 1:**
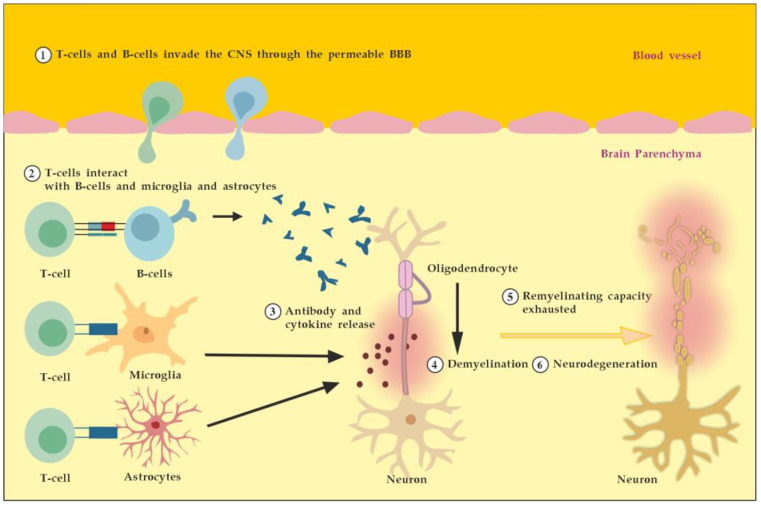
Pathogenesis of MS. Described processes 1 to 6 occur consecutively and in parallel in terms of the spatial (peripheral vs. CNS inflammation) and temporal (acute vs. chronic inflammation) axes. Adapted from [3] and modified.

MS is an autoimmune multifocal CNS inflammatory disease, characterized by chronic inflammation, demyelination, axonal damage, and subsequent gliosis. The most affected CNS regions by the disease are the periventricular area, subcortical area, optic nerve, spinal cord, brainstem, and cerebellum. MS is categorized as relapsing–remitting (RR), secondary progressive (SP), and primary progressive (PP) [11]. The pathogenesis of MS suggests that in genetically susceptible subjects, independent populations of T lymphocytes are activated in the immune system, migrate across the BBB, and trigger CNS tissue damage. They release pro-inflammatory cytokines, initiate cytotoxic activities of microglia with the release of nitrous oxide and other superoxide radicals, stimulate B cells and macrophages, and activate the complement system [12]. Autoantibodies against myelin basic protein and myelin OLs glycoprotein have been detected in MS patients. These antibodies may mediate injury by complement fixation or linking with innate effector cells such as CNS resident macrophages [12]. Despite the specific clinical form, the initial stage of the disease is characterized by an autoimmune inflammatory response mainly against the OLs in the CNS, resulting in demyelination (inflammatory component). Soon after the regenerative capacity of the OLs is exhausted, the inflammatory processes attack the neurons themselves, leading to the permanent injury and dysfunction of the CNS (neurodegenerative component). Both inflammatory and neurodegenerative components of MS pathogenesis are believed to be involved from the very beginning of the disease, giving different clinical presentations in the context of spatial and temporal pathological changes) (Figure 1) [13].

### 3.2. Between Detrimental and Beneficial Neuro-Inflammatory Responses

#### 3.2.1. The Role of Peripheral Immune Cells

**Figure 2:**
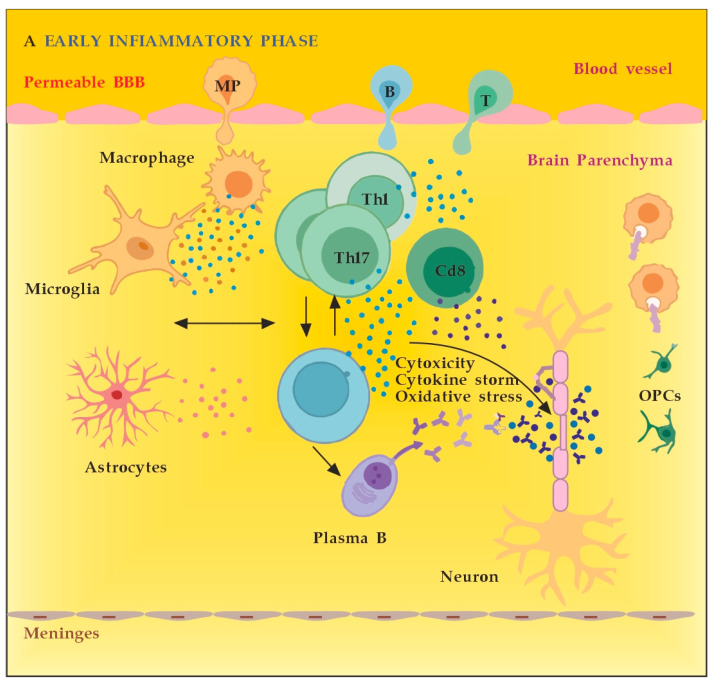

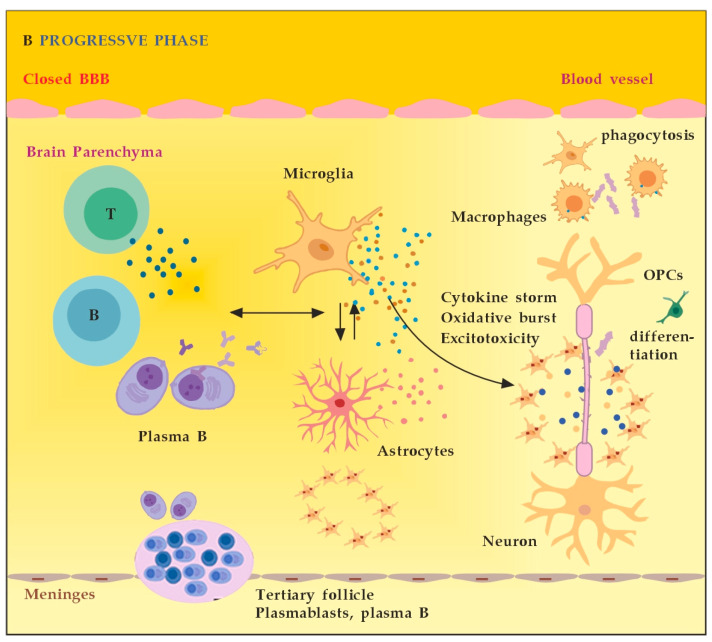
(**A**) The role of peripheral immune cells in MS. Adapted from [18] and modified. (**B**) The role of peripheral immune cells in MS. Adapted from [18] and modified. [dots: in blue and purple (cytokines and cytotoxic products from adjacent cells); in brown (reactive oxygen species)].

MS neuro-inflammation is characterized by pathogenic immune responses involving T cells (CD4+ and CD8+ T cells), B cells, and myeloid cells along with the reduced function of regulatory T cells [14]. In the early inflammatory phase of MS, peripheral adaptive immune cells infiltrate the CNS through a compromised BBB (Figure 2A). These activated cells interact with each other and with CNS resident cells. They secrete cytokines such as IFN-γ by Th1, IL-6, IL-17 by Th17, GM-CSF, IL-6, TNF-α by B cells, and cytotoxic molecules such as granzyme B by CD8+ T cells. B cells can further evolve into pathogenic autoantibody-producing plasma cells. As a result, T and B cells activate macrophages and microglia that produce cytokines, nitric oxide, and reactive oxygen species (ROS). This cytotoxic pro-inflammatory environment causes oligodendrocyte and axonal damage through direct cell contact-dependent processes and the release of neurotoxic mediators [13]. It destroys the myelin sheaths around axons and causes energy failure in the axon. Yet, macrophages and microglia can still clear the myelin debris, allowing for the recruitment of oligodendrocyte progenitor cells (OPCs) that will partially remyelinate the lesion [13,15].

In the progressive phase of MS, the inflammation is restricted within the CNS due to the persistence of activated immune cells in situ, despite the absence of infiltrating T and B lymphocytes from the periphery (Figure 2B). This chronic inflammatory process affects the whole brain parenchyma, even at sites distant from the underlying focal demyelinating lesions. Diffuse chronic CNS inflammation is thought to be more common in patients with progressive forms of MS [16,17]. Notably, plasmablasts and plasma B cells form tertiary follicle-like structures in the meninges. Their location in the leptomeninges contributes to the demyelination of subpial gray matter and highlights the importance of B lymphocytes in the pathogenesis of the progressive form of MS. The BBB is closed and the inflammation is maintained by innate resident CNS cells, i.e., microglia and astrocytes. They produce cytokines (TNF-α, IL-6) and release ROS, causing damage to myelin [18].

Recent data suggest that neuro-inflammation may be beneficial to some extent [19]. In experimental autoimmune encephalomyelitis (EAE) models, the treatment of mice with IFN-γ, classically considered a pro-inflammatory cytokine, leads to reduced morbidity and mortality [20]. Evidence also supports the protective role of tumor necrosis factor alpha (TNF-α) in EAE. Mice lacking TNF-α and its related receptors showed a significant delay in remyelination [21]. This could be related to the missing TNF-α induction of neurotrophins expression as well, such as NGF and brain-derived neurotrophic factor (BDNF) [22]. TNF-α treatment significantly reduced the severity of the disease in immunized TNF-deficient mice [23]. The results suggest that some pro-inflammatory cytokines may also play an indirect rather than direct role in disease control and remyelination [20,21,23]. Anti-inflammatory cytokines, such as IL-4 and IL-10, may have a direct protective effect instead [24,25].

Immune cells also exert a neuroprotective effect in MS via the production and local secretion of neurotrophins, such as NGF and BDNF [26]. In addition, after suppressing B cell cytokines BAFF and APRIL with atacicept (cytokines important for B cell survival and function), adversely increased clinical activity in MS was observed. The latter provides indirect evidence for the anti-inflammatory functions of certain B cells [27].

#### 3.2.2. The Role of the Innate Resident CNS Cells

The main innate resident CNS cells of relevance to the localized inflammatory response are microglia and astrocytes. They produce cytokines (TNF-α, IL-6) and release ROS in situ, causing myelin damage [28].

**Figure 3:**
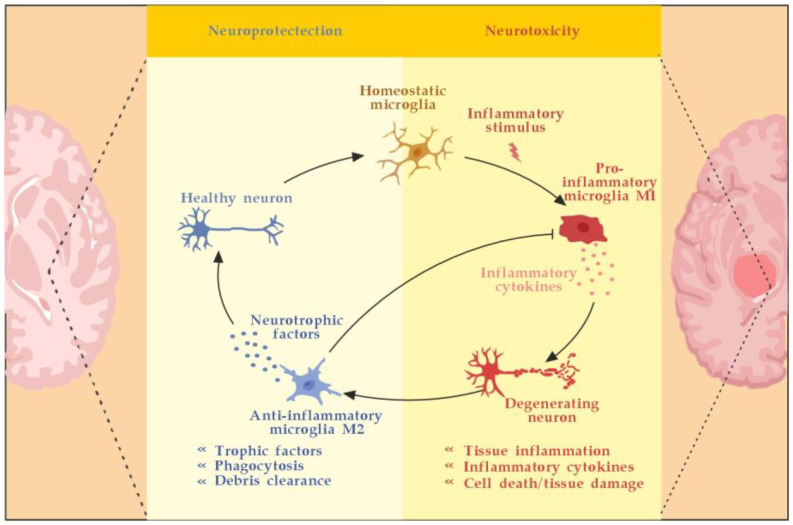
Microglia activation and resolution of inflammation. Adapted from [46] and modified.

Microglia are activated by pathogen-associated molecular pattern molecules (PAMPs) and/or damage-associated molecular pattern molecules (DAMPs) [29]. The classical (M1) microglia activation produces pro-inflammatory cytokines and chemokines, such as tumor necrosis factor-alpha (TNF-α), interleukin (IL)-6, IL-1β, IL-12, and CC chemokine ligand 2 (CCL2), and induce inflammation and neurotoxicity) [30]. The alternative (M2) microglia activation secretes certain growth factors (GFs) and neurotrophic factors (NTFs) and promotes the survival of neurons [31]. The switch from M1 to M2 phenotype may occur via inhibition of nuclear factor kappa-light-chain-enhancer of activated B cells (NF-κB), mitogen-activated protein kinase (MAPK), activator protein 1 (AP-1), and signal transducer and activator of transcription (STAT) transcription factors, and activation of the peroxisome proliferator–activated receptor gamma (PPARγ) pathway [31,32,33,34,35,36] (Figure 3).

Microglia activation contributes to MS disregulation through antigen presentation, secretion of pro-inflammatory cytokines, and phagocytic processes [37,38]. Although microglia are primed into a pro-inflammatory phenotype, their phagocytic capacities are diminished. Myelin debris are not completely cleared, OPCs are insufficiently recruited and fail to differentiate. Overall, microglial activation in the CNS is heterogeneous and cannot be classified only into two different subtypes: classical (M1) or alternative (M2) [39]. Although M1 microglia promote inflammation and M2 microglia have an anti-inflammatory phenotype, there appears to be a continuum of phenotypes between M1 and M2 that can switch from one to another [40]. Modulation microglia M1/M2 polarization and shifting from M1 to M2 phenotype have been proposed as promising therapeutic strategies in neurodegenerative CNS disorders [41].

Astrocytes may play a role in inhibiting remyelination and axonal regeneration through reactive astrogliosis, glial scar formation, and the secretion of inhibitory molecules which suppress axonal growth [42]. TNF-α-mediated glutamate release from astrocytes leads to excitotoxicity, causing axonal damage. The ferrous iron (Fe^2+^) released from the myelin is oxidized to produce ROS leading to a major oxidative burst, causing mitochondrial dysfunction, mitochondrial DNA damage, energy failure, and axonal loss [18].

Activated astrocytes show a Janus-faced nature. The A1 astrocytes secrete interleukin (IL)-1β, TNF-α, and C3 components to propagate the neuroinflammatory response. Additionally, they also secrete D-serine and nitric oxide (NO), which may contribute to excitotoxicity with subsequent neuronal and oligodendrocyte death. The alternative A2 astrocytes, however, may secrete anti-inflammatory compounds, such as neurotrophic factors (NTFs, including NGF), IL-10, IL-6, and TGF-β, and promote the neuroprotective and neuroregenerative functions [41,43].

Astrocytes may stop the T-cell response by inducing apoptosis as well [44]. Until recently, astrocytes’ formation of the glial scar was considered a harmful process that impedes the regeneration and remyelination of axons. Seen from a different perspective, however, depending on the severity of the injury, the scarring process may also serve to isolate the inflamed area, provide structural support, and restrict damage [42]. Likewise, activated microglia can also promote remyelination by clearing myelin debris from the local environment and by secreting anti-inflammatory cytokines, such as transforming growth factor-beta 1 (TGF-ß1) and certain neurotrophic factors, such as BDNF, that can induce the proliferation of OLs (Figure 3) [45,46].

## 4. The Role of Autoantibodies

B cells and their evolving plasma cells, along with the plasma cells producing autoantibodies and complement, have been found in MS lesions [47], indicating their implication in demyelination. Further evidence for antibody-mediated mechanisms in MS comes from the presence of ectopic lymphoid follicles in the CNS of MS patients [48], particularly those with progressive disease. The remarkable success of B-cells depleting therapies suggests that plasmablasts and plasma cells-producing autoantibodies may promote deterioration of the disease. However, the ability of plasma cells and their autoantibodies to significantly affect the course of MS is still a matter of debate [49].

Until recently, the available clinical and experimental evidence suggested that no specific antibodies had been identified in MS [50]. Pathological studies have shown IgG and complement deposition in brain lesions in some MS cases (defined as type II lesions), suggesting the contribution of humoral immunity and autoantibodies to the pathogenesis [51]. In confirmation of these data, some authors reported a subset of patients with type II MS lesions that had autoantibody-induced demyelinating responses [52,53]. Experimental research revealed that MS myelin-specific IgG1 monoclonal recombinant antibodies initiate complement-dependent cytotoxicity to OLs (oligodendrocyte loss) and induce rapid demyelination. The research gives compelling evidence of antibody/complement contribution to the type II MS lesions given the deposition of IgG and activated complement in EAE model. Importantly, antibody-induced demyelination was accompanied by significant activation of microglia [54]. In a human study, serum antibodies against the cytoplasm of OLs were detected in a relatively small proportion of MS patients with primary or secondary progressive disease. Compared to anti-oligodendrocyte autoantibody-negative MS patients, anti-oligodendrocyte antibody-positive MS patients were significantly older at the time of serum sampling, showing significantly greater impairment (significantly higher Kurtzke EDSS scores) and a higher frequency of mental disorders [55]. Another human study demonstrates that myelin obtained post mortem from MS brain donors is bound by IgG antibodies. In addition, IgG immune complexes strongly potentiate the activation of primary human microglia, leading to increased production of key pro-inflammatory cytokines, such as TNF and IL-1b. Thus, IgG immune complexes and activated human microglia may play an increasing role in MS-related inflammation and demyelinating lesion formation [56]. Most recent experimental research revealed that MS plasma IgG antibodies form large aggregates (>100 nm) that generate complement-dependent apoptosis in neurons and astrocytes. These findings provide a direct link between IgG antibodies and neuron death [57]. In real-world clinical practice, with the development of a novel nanomembrane-based TPE technology (with membrane pores 30–50 nm diameter [58]), by removing these pathological antibodies and immune complexes, we could modulate not only the demyelination (oligodendrocyte loss) and microglial activation but the neuronal and astrocyte apoptosis as well [54,55,56,57]. This modulation could have a significant impact on the levels of synthesized NGF by OLs, astrocytes, and neurons, which will be discussed.

In the early stage of MS of inflammatory demyelination, increased levels of monosialoganglioside 1 (GM1), the main myelin ganglioside, were found [59]. In addition, anti-ganglioside antibodies were observed that could either contribute to axonal degeneration or appear as a consequence of axonal damage. Whatever is true does not change their potential to cause BBB disruption [60] and inhibition of axonal regeneration [61]. Given the fact that the deleterious effect of anti-ganglioside IgM antibodies on BBB leakage is concentration-dependent but complement-independent [62], the plasma removal of these primary or secondary autoantibodies would also be pathogenetically reasonable.

**Figure 4:**
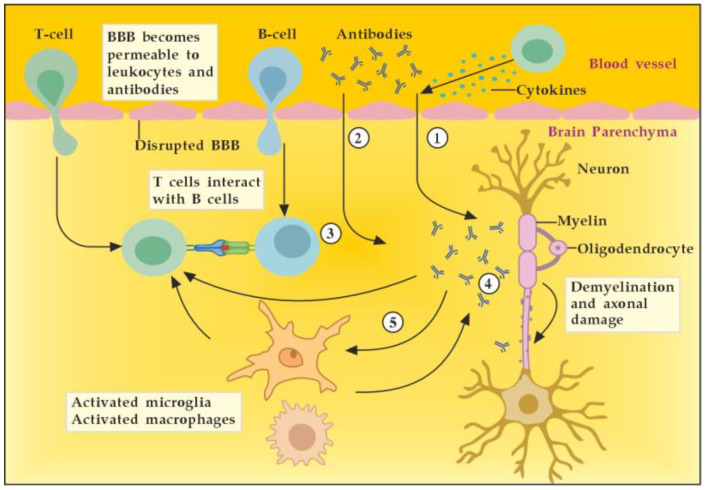
Potential mechanisms of antibody pathogenicity in multiple sclerosis. Adapted from [7] and modified. [activated T cells ① and circulating antibodies ② damage BBB and increase its permeability; activated B cells ③ add to the antibodies production in brain parenchyma; antibodies induce direct damage ④ or promote demyelination indirectly via activation of microglia and macrophages ⑤].

In general, we could outline the pathogenicity of circulating autoantibodies associated with MS by several different possible mechanisms of actions and interactions (Figure 4). For example, during the early inflammatory phase due to increased BBB permeability induced by encephalitogenic T cells, circulating antibodies can reach the brain and become pathogenic. In addition, these antibodies themselves could induce vascular damage and inflammatory lesions of the CNS by complement-dependent or antibody-dependent cellular cytotoxicity mediated through Fc receptors on microglia and macrophages. Moreover, autoreactive B cells could infiltrate the brain and induce high levels of pathogenic autoantibodies in the cerebrospinal fluid (CSF). In the brain parenchyma, autoantibodies bound to the surface of target cells could cause their direct damage or functional alteration, which in turn leads to demyelination. Finally, autoantibodies may also promote demyelination indirectly by activating autoreactive T cells or microglia and macrophages [7]. By removing the pathogenic antibodies from the circulation and CNS when BBB permeability is available, we can modulate all described mechanisms and pathological consequences of antibodies’ actions and interactions.

## 5. The Role of NGF

**Figure 5:**
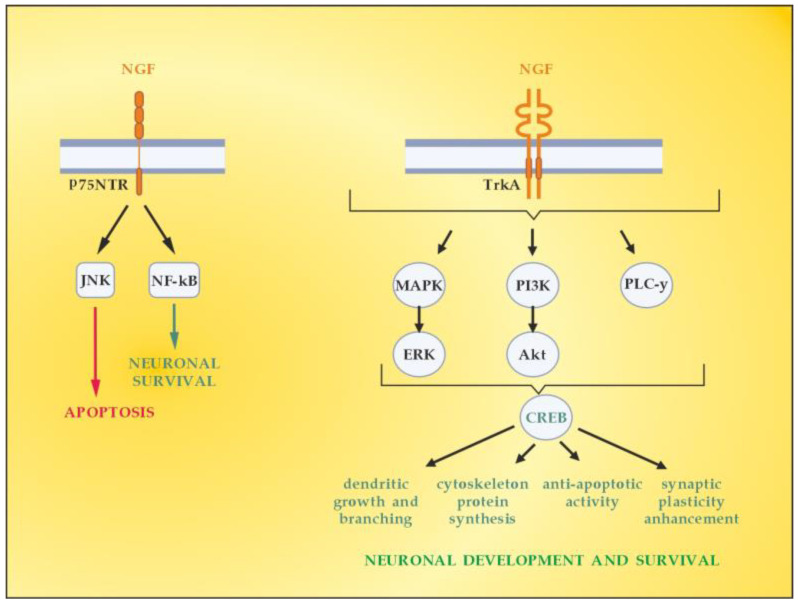
Signaling pathways activated by NGF. Adapted from [41] and modified.

NGF is the member of the neurotrophin family which has been described in 1952 by Levi-Montalcini [63]. The neurotrophin family also includes neurotrophin-4 (NT4), BDNF, and neurotrophin-3 (NT-3) [64]. NGF activates downstream signaling cascades by binding to two types of membrane receptors, TrkA and p75NTR [65]. TrkA is considered a high-affinity receptor that selectively binds the NGF, thus conveying pro-survival signals [66]. p75NTR belongs to the tumor necrosis factor receptor family (TNFR) binding to all mature neurotrophins with the same affinity, while being considered a high-affinity receptor for the immature isoforms of the neurotrophins, the so-called pro-neurotrophins [67]. Depending on the NGF-specific receptors (high-affinity TrkA and low-affinity p75NTR), signaling pathways for neuronal differentiation, maturation and survival, axonal and dendrite development, or apoptosis can be triggered (Figure 5). The p75NTR forms complexes with various receptors, thus mediating a great number of different and sometimes opposing functions, the latter depending on the cellular and environmental context [68]. When TrkA and p75NTR are co-expressed, they constitute a two-receptor heterotetrameric system that binds to NGF and activates various signaling pathways [69,70].

In the context of demyelinating damage that accompanies MS development, it is essential to emphasize the role of neurotrophins (including NGF) in the activation of the principal signaling pathways driving OLs differentiation, myelination, and corresponding remyelination after injury, namely, Erk1/2-MAPK (Figure 5) [71]. The mitogen-activated protein kinase (MAPK) pathway by extracellular signal-related kinases 1 and 2 (Erk1 and Erk2) is reported to regulate oligodendroglial development, proliferation, apoptosis, differentiation, and myelination [72,73]. The Erk1/2-MAPK pathway is activated by platelet-derived growth factor (PDGF) and neurotrophins (NGF, NT3, and BDNF). This triggers a cascade of processes involving phosphorylation of MAP3K, MEK1, and MEK2, as well asErk1 and Erk2, which are finally translocated to the cellular nucleus [74,75]. Once there, they can regulate the expression of the OLs transcription factor myelin regulatory factor (MYRF), which promotes remyelination in MS [76].

NGF is a neurotrophin that is largely expressed in the CNS in neurons, OLs, and astrocytes as well as in the periphery [77,78]. NGF and its receptors are expressed by almost all different cell types, including T cells, B cells, macrophages, neutrophilic and basophilic granulocytes, mast cells, etc. (Table 1) [79,80]. NGF could affect B cells (proliferation, immunoglobulin production, and cell proliferation), T cells (survival and expression of cytokine receptors) and plays a special role in macrophage antigen presentation and migration into inflamed regions and lesions [81,82]. What is more, NGF activates chemotaxis [83,84,85,86], stimulates the phagocytosis of neutrophils [87] and macrophages [84,85], increases the cytotoxic activity of eosinophils [88], and stimulates the degranulation of mast cells [89,90].

In the CNS, NGF specifically provides trophic support to cholinergic neurons of the basal forebrain that express TrkA, which makes it particularly interesting for Alzheimer’s disease (AD) [91,92,93,94,95]. NGF and its receptors, TrkA and p75, are reported to play a bi-directional role between the immune and nervous systems. NGF plays a dual role both in anti- and pro-inflammatory responses [96]. At the site of inflammation, pro-inflammatory cytokines (such as IL-1β and IL-6) induce overexpression of NGF [97]. p75NTR, in the absence of the TrkA co-receptor, can affect the migration of B cells via BBB, where it has been described to play a crucial role, as well as limit the production of autoantibodies from B cells. By this mechanism, NGF performs neuroprotection in the context of protective autoimmunity, where the organism develops specific mechanisms to cope with CNS damage by restricting and controlling degeneration and/or promoting regeneration [98,99]; and vice versa, NGF using TrkA could upregulate axonal expression of LINGO-1 (a membrane-bound protein, part of the Nogo-A signaling pathway and a myelin-associated inhibitor) and may also negatively affect the process of axonal myelination [100,101]. However, the deletion of Nogo-A signaling (using Nogo-A knockouts animal model) fails to maintain regeneration of axons after spinal cord injury. Hence, the Nogo-A/LINGO1 signaling pathway may not play an important role in the failure of regeneration but instead could participate in an accessory function [102]. The latest research corroborates the regenerative ability of NGF using adipose mesenchymal stem-cell-derived NGF. After injecting into the animals with EAE, the artificial NGF stimulates axon regeneration and also decreases neurogliosis [103]. Moreover, another recent study using an in vitro model of mixed neural stem-cell-derived OPCs revealed that in the mixed culture, astrocytes are the major producer of NGF, and OPCs express both TrkA and p75NTR. NGF treatment increases the percentage of mature OLs, whereas NGF blocking by neutralizing antibodies impairs OPC differentiation. This report clearly demonstrates that NGF is involved in OPC differentiation, maturation, and protection, which also suggests possible implications in the treatment of demyelinating lesions and related diseases [104].

In the course of neuro-inflammation, almost all resident CNS cells overexpress NGF [105]. In addition, NGF in the blood could cross the BBB and reach glial cells, when BBB becomes permeable under pathological conditions, such as, for example, MS [106]. It should be pointed out that NGF levels affect glial physiology. As reported in in vivo mouse model [107], the reduction of NGF leads to A1 activation of astrocytes and neurotoxicity. On the contrary, as previously reported [108], the elevation of NGF steers microglia toward an anti-inflammatory phenotype, thus leading to neuroprotection. In addition, previous research observed the significant effects of intracranial administration of NGF on cytokine expression, which were specific for the CNS parenchyma and were not found in the periphery [109]. As far as MS is concerned, during acute attacks, patients show elevated levels of NGF in the CSF compared with healthy individuals, which can be regarded as an attempt to protect the CNS tissue against inflammation [110]. All these findings suggest the relevance of NGF-based therapeutic approaches in cases of inflammatory and neurodegenerative disorders [107].

The role of NGF in modulating the activity of a number of cellular and tissue structures during CNS inflammation and injury is presented in Table 2.

Recently, it has been shown that TNF-α not only induces NGF over-expression but modulates the NGF signaling pathways as well. The cross talk between these two is possible due to the fact that p75NTR belongs to the tumor necrosis factor receptor family (TNFR). TNF-α downregulates the mRNA and protein levels of TrkA and also increases p75 mRNA expression [110]. This could shift the role of NGF signaling from neuroprotective to neurotoxic, implying that a specific binding of a certain receptor is of significant importance, especially during inflammation [110]. In turn, NGF can modulate the TNF-α signaling pathways by downregulating TNFR1-mediated apoptosis and promoting preferential signaling through TNFR2, which leads to protection and proliferation. What is more, NGF also induces production of BDNF [22], another CNS neurotrophin and well-established activator of re-myelination in MS [101].

Finally, NGF antibodies were observed to exacerbate neuropathological signs of EAE [111]. This implies not only the significance of NGF in reducing the extent of EAE lesions [112] but also opens up a new possibility to enhance the NGF beneficial anti-inflammatory potential in MS patients by removing these antibodies by means of TPE [113].

## 6. The Role of S1P

Sphingolipids are functionally active participants in a wide range of extracellular and intracellular processes [114,115]. The balance between sphingosine and sphingosine-1-phosphate (S1P), both being metabolites of the precursor ceramide, and their subsequent phosphorylation by enzymes called kinases were shown to be important in the determination of whether a cell is destined for cell death/apoptosis or proliferation [116]. Although S1P is essential for normal CNS development and maturation [117] and it also may regulate synaptic function [118], it can also have cytotoxic effect at higher concentrations, such as when there is a genetically determined deficiency in its degradative enzymes [119]. S1P also regulates calcium metabolism [120] and may promote presynaptic calcium overload and eventually cell death [121]. Of note, S1P is implicated in both upstream and downstream production of cytokines, and increased interstitial levels of S1P at the inflammatory sites induce the expression of pro-inflammatory cytokines [122]. As described above, free interstitial S1P increases at inflammation sites, where, unlike its plasma anti-inflammatory effects, this sphingolipid is involved in the propagation of inflammation [122].

A distinguishing characteristic of the members of the sphingolipid family is their participation in pro- or anti-proliferative pathways of cell regulation [114]. Especially, S1P is well known for its wide functional activity, influencing processes such as cellular migration, adhesion, differentiation, and survival, among others. It is also an active participant in the genesis of various pathological processes and diseases, involving inflammation, oxidative stress, neurodegenerative pathologies such as MS, etc. [123]. MS is an autoimmune inflammatory neurodegenerative disease, which is characterized by disturbances in the sphingolipid metabolism in the CNS [123]. The levels of S1P are reported to be elevated in the cerebrospinal fluid of MS patients, and this elevation shows specific correlations with the clinical severity of the disease (e.g., Kurtzke EDSS score) [124]. The high concentrations of S1P occurring in cerebrospinal fluid [125] from MS patients support the presumption that the bioactivity of S1P is pro-inflammatory rather than protective [123]. In addition, S1P affects the integrity of the BBB, which is generally damaged in patients with MS [123].

**Figure 6:**
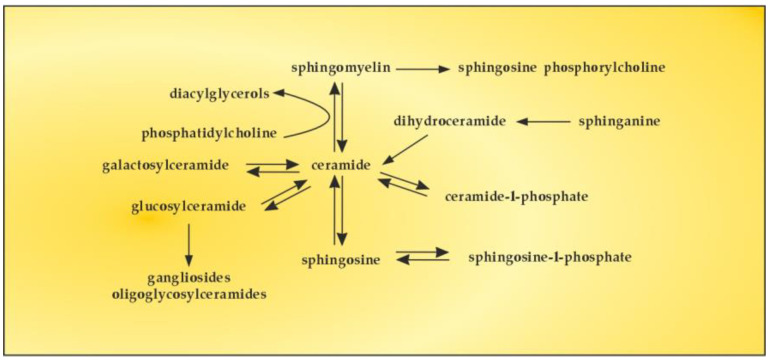
The SM–S1P pathway.

The bioactive lipid, S1P, is generated by phosphorylation of sphingosine, catalyzed by two isoforms of sphingosine kinase (SK1 and SK2). S1P can also be reversibly dephosphorylated by S1P phosphatase to produce sphingosine, the levels of which are generally controlled by flux through de novo ceramide synthesis and sphingosine catabolic pathways [126]. There are numerous studies on the sphingomyelin (SM)–S1P pathway in order to reveal whether SM serves as a major source of S1P through the activities of sphingolipid metabolizing enzymes [127]. The obtained results showed upregulation of certain sphingolipid catabolizing enzymes, implying that SM could serve as a possible source of S1P (Figure 6).

A vast number of the biological effects of S1P are mediated by a family of G-protein-coupled S1P receptors S1P1–S1P5. The complex expression patterns and transmembrane and intracellular signaling pathways of each receptor form the molecular basis for the diversity of S1P functions [122].

S1P receptors are widely expressed in cells of the CNS [128], including neurons, astrocytes, microglia, and OLs, all of them having potential roles in the pathogenesis of MS. S1P1 upregulates Th17 polarization and increasing neuro-inflammation, which are key factors in MS pathogenesis [129]. During inflammation, an S1PR-1-dependent upregulation of microglia is observed which additionally increases the inflammatory process [130]. The blocking of S1P1 decreases activated microglial production of pro-inflammatory cytokines (TNF-α, IL-1β, and IL-6) and increases production of BDNF and glial-cell-derived neurotrophic factor, the latter being neuroprotective [131]. In addition, S1PR-1 blockade is a potentially important pharmacological target to reduce astrogliosis and promote re-myelination in MS patients [132]. A role of S1P1 in astrocytes has been shown in the disease progression [133]. Both S1PR-1 and S1PR-3 are upregulated by pro-inflammatory astrocytes and are associated with higher production of glial acidic fibrillary protein [134]. The potential of S1P2 to destabilize adherent junctions, promote inflammation, and modulate the infiltration of leukocytes may increase the disease severity [133]. As far as S1P3 signaling in MS is concerned, its actual sequelae regarding detrimental effects (e.g., astrogliosis) and beneficial effects (e.g., remyelination) could not be determined [135]. Clearer evidence for the pro-inflammatory contribution of S1P3 was reported later [136].

Red blood cells (RBCs) and endothelial cells (ECs) are major sources for the production of plasma S1P. About 50–60% of the circulating S1P is bound to apolipoprotein M (ApoM)/high-density lipoprotein (HDL), and 30–40% is bound to albumin. Platelets may also participate in the production of plasma S1P, especially upon platelet activation, which significantly enhances S1P release [137]. Experience from the COVID-19 pandemic implies that during severe inflammation the decrease in S1P is closely connected to the number of RBCs, the major source of plasma S1P, and to ApoM/HDL and albumin, the major transporters of S1P in blood [138].

Alterations in blood flow modulate endothelial S1P secretion and receptor S1P1 expression. In static state, there is a decrease in S1P production and secretion of endothelial S1P. In addition, S1P1 transcription is less active, which leads to lower S1P1 activity. On the contrary, in active state, shear stress substantially upregulates S1P1 expression, which induces an increase in endothelial S1P levels and enhanced S1P1 signaling. S1P enzymatic degradation in tissues is a key factor in the formation of circulatory S1P gradient across the endothelial barrier, which keeps S1P levels at ~1 μM in the blood, at ~0.1 μM in the lymph, and at <1 nM in the interstitial fluids [137].

The above determinants of circulatory S1P gradient along with the S1P plasma levels could be modulated during TPE, which will be discussed later.

## 7. The Role of TPE

TPE is an invasive therapeutic method that involves extracorporeal blood removal, as well as the return or exchange of blood plasma or components. It usually removes a large volume of plasma (1 to 1.5 of patient’s total plasma volume (TPV) per treatment) with adequate volume replacement using colloid solutions (e.g., albumin and/or fresh frozen plasma (FFP)) or a combination of crystalloid/colloid solutions [139]. TPE is applied to remove pathogenic substances with high molecular weight (>150 kDA) including autoantibodies, immune complexes, pro-inflammatory mediators, lipids, and many others from the intravascular space, which ensures its rapid onset of action [139,140]. However, the mechanism of action of TPE in immune-mediated inflammatory and neurodegenerative disorders involves more than the simple removal of large pathogenic molecules. For example, the application of TPE may also modulate cellular immunological response by altering the ratio between T-helper type-1 (Th-1) and type-2 (Th-2) cells in peripheral blood. Th-2 cells maintain the humoral immune response by facilitating B-cell autoantibody production and play an essential role in neurodegenerative autoimmune disorders. By shifting the balance between peripheral T cells from Th-2 predominance to Th-1 predominance, TPE has a modulatory effect on the pathogenic immune response and may play a therapeutic role within and beyond the time of TPE application [141].

**Figure 7:**
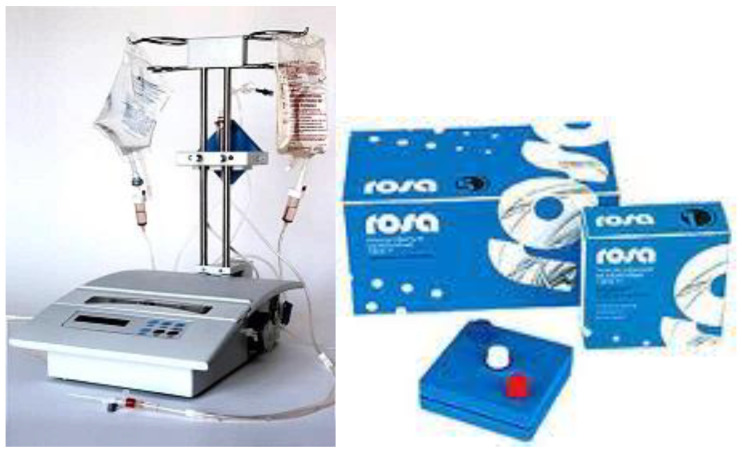
Hemophenix apparatus with ROSA nanomembrane [58].

**Figure 8:**
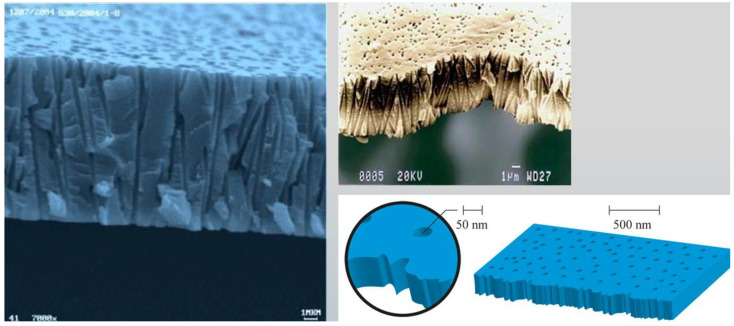
Electron microscopic profile of the track membrane ROSA [58].

The contemporary status of TPE in autoimmune neurological diseases in Japan suggests that it can be considered as an efficient therapy for autoimmune neurological diseases such as MS, myasthenia gravis (MG), neuromyelitis optica spectrum disorders (NMOSD), chronic inflammatory demyelinating polyneuropathy (CIDP), and Guillain–Barré syndrome (GBS), among others, with a low frequency of adverse effects [142]. Our data corroborate these findings in the mentioned neurological disorders after the use of nanomembrane-based TPE [58]. This innovative approach involves passing the patient’s blood through several nanomembranes, aiming to filter certain large molecules [113,143,144,145,146,147,148]. The nanomembrane-based technology involves the use of the “Hemophenix” apparatus (Figure 7) with the ROSA nanomembrane (“Trackpore Technology”, Moscow, Russia) (Figure 8). The nanomembrane has pores with a diameter of 30–50 nm, and it can filter molecules with molecular weights less than 40 kDa. The device has a filling volume of up to 70 mL and also possesses the advantage of a single-needle access to a peripheral vein [149].

**Figure 9:**
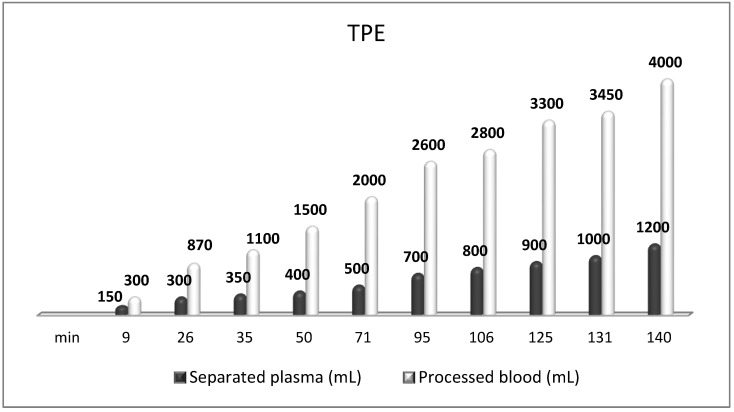
Amount of processed blood and separated plasma (LVPE) during a TPE procedure in a patient with MS.

The most frequently used replacement fluid in nanomembrane-based TPE is saline (NaCl 0.9), which has low cost and no adverse effects, even when 25% (approximately 700–750 mL plasma) of the circulating plasma is removed [144]. Our practice of saline replacement in the removal of 700–750 mL of plasma is in agreement with the so-called low-volume plasma exchange (LVPE), which ranges from 350 mL to 2 l plasma volume removal per each separate procedure. The LVPE approach is preferred in chronic conditions, in which the separation of smaller volumes of plasma would be justified for long periods of time [150]. The relevance of minimizing the adverse events of colloid replacement by lowering plasma volume exchanged per treatment (0.5–0.7 of TPV) is supported by the German practice in the field of LVPE as well [151,152]. The reported data suggest that effectiveness may be provided with volumes below the currently recommended volumes (1 to 1.5 of TPV) [151,153]. According to the Spanish practice, the LVPE approach suggests good effectiveness in neuro-immunological disorders (GBS, NMOSD, MG, MS). However, more profound studies are needed to confirm LVPE as a better alternative to the classical TPE [150]. Nevertheless, our experience in LVPE adds new insights concerning the effectiveness of the low-volume approach after implementing an innovative nanomembrane-based technology (Figure 9).

**Figure 10:**
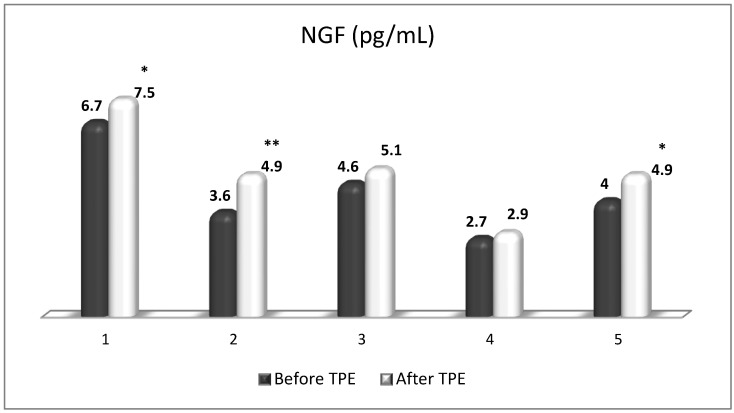
Changes in the level of NGF before and after a TPE procedure in patients (1, 2, 3, 4, 5) with acute exacerbations of relapsing–remitting MS (* *p* < 0.05; ** *p* < 0.01).

**Figure 11:**
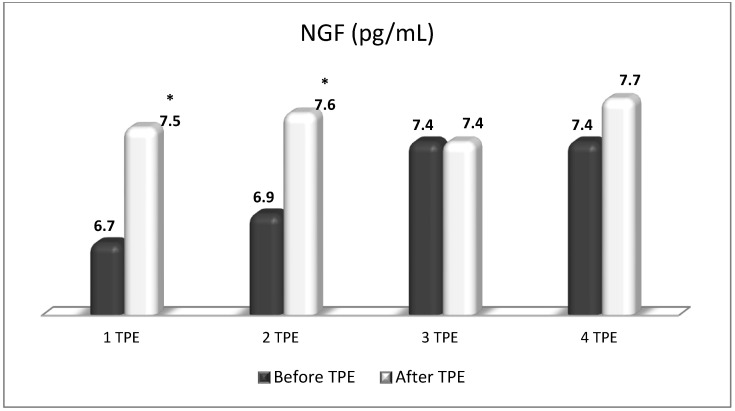
Changes in the level of NGF before and after the course of TPE procedures (1, 2, 3, 4) in a patient with acute exacerbations of relapsing–remitting MS (* *p* < 0.05).

**Figure 12:**
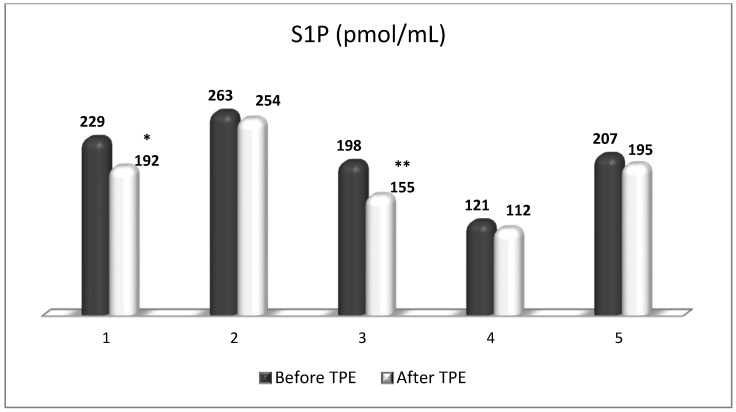
Changes in the level of S1P before and after a TPE procedure in patients (1, 2, 3, 4, 5) with acute exacerbations of relapsing–remitting MS (* *p* < 0.05; ** *p* < 0.01).

**Figure 13:**
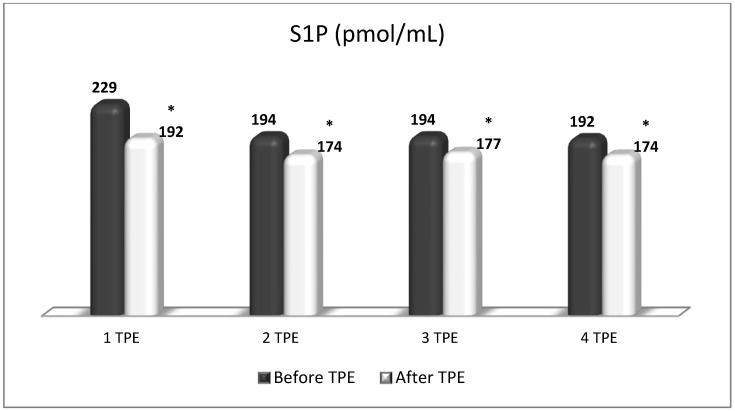
Changes in the level of S1P before and after the course of TPE procedures (1, 2, 3, 4) in a patient with acute exacerbations of relapsing–remitting MS (* *p* < 0.05).

**Figure 14:**
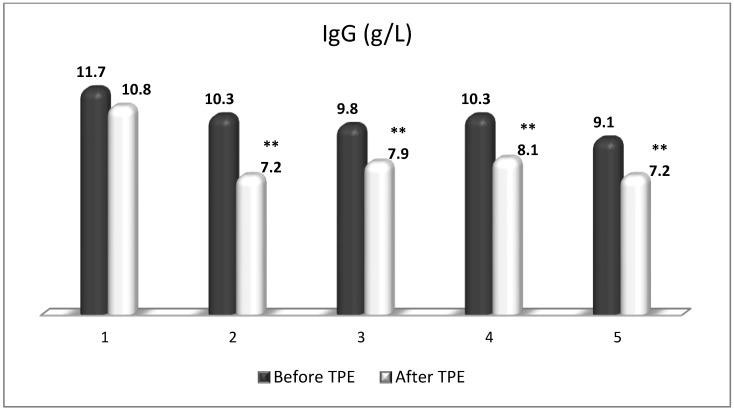
Changes in the level of IgG before and after a TPE procedure in patients (1, 2, 3, 4, 5) with acute exacerbations of relapsing–remitting MS (** *p* < 0.01).

**Figure 15:**
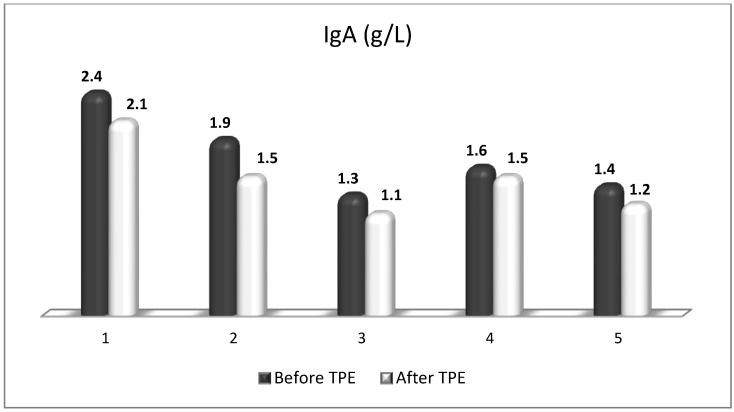
Changes in the level of IgA before and after a TPE procedure in patients (1, 2, 3, 4, 5) with acute exacerbations of relapsing–remitting MS (*p* > 0.05, there is a trend towards reduction).

**Figure 16:**
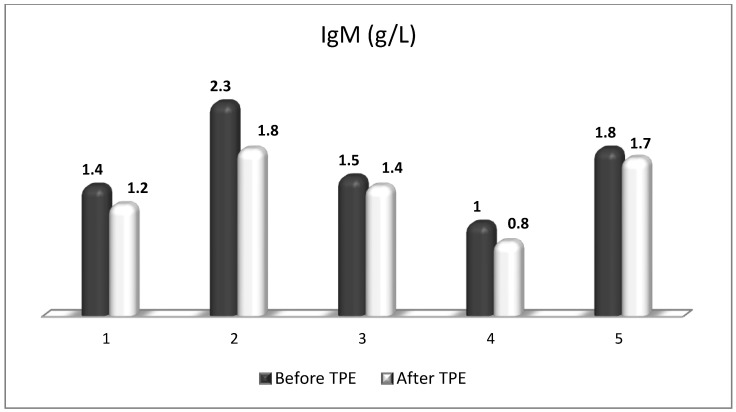
Changes in the level of IgM before and after a TPE procedure in patients (1, 2, 3, 4, 5) with acute exacerbations of relapsing–remitting MS (*p* > 0.05, there is a trend towards reduction).

TPE is presumed to affect NGF and S1P plasma levels in many different ways. In classical filtration TPE (1–1.5 of TPV), membrane pores might be blocked by red blood cells, and hemolysis may occur depending on the hematocrit and the blood flow rate or blood shear rate [154]. In addition, the shear flow and shear stress are factors that affect the leukocyte-material-induced activation [155]. Thus, strict control of the transmembrane pressure is required [154] in order to avoid these TPE-associated adverse effects. In LVPE (0.5–0.7 of TPV), the ratio surface area/plasma volume is more favorable in terms of minimizing hemolysis and leukocytes’ activation [156]. This is likely to affect the plasma levels of NGF and S1P. In our clinical settings, the changes in hemoglobin (a major source of S1P synthesis [135]), albumin, and ApoM/HDL (major carriers of circulating S1P [137]), after administering albumin/FFP replacement fluids (infusion of albumin/FFP stimulate the release of S1P from erythrocytes and platelets [157]), activated leukocytes (source of growth factors [158]) and are all balanced by the use of nanomembrane-based LVPE. Beyond these considerations, the most plausible explanation for the observed elevation of NGF plasma levels (Figure 10 and Figure 11) and reduced S1P plasma levels (Figure 12 and Figure 13) in our cases of MS patients could be due to the removal of autoantibodies (Figure 14, Figure 15 and Figure 16) against NGF-producing cells and NGF itself (discussed above) as well as direct loss of S1P with discarded plasma [159]. The reduction of blood S1P after TPE leads to the inability to maintain the circulatory S1P levels and to the accumulation of mature T cells in lymphoid organs [160]. This may have the same clinical implications for MS patients as the administration of S1P1 receptor modulator fingolimod, causing lymphocyte sequestration in peripheral lymphoid organs and thus preventing autoreactive immune cells’ infiltration into the CNS [161]. The increased NGF plasma levels after TPE application could either contribute to or occur as a consequence of increased NGF levels in the CNS (given NGF’s ability to cross the permeable BBB according to its gradient [106]). In both cases, they should be considered as a favorable anti-inflammatory response as a result of the reduced pro-inflammatory load related to discarded plasma. The augmented NGF in CNS could steer glia toward an anti-inflammatory phenotype and neuroprotection [108]. Likewise, the removal of circulatory pathogenic factors (autoantibodies, immune complexes, cytokines, etc.) from peripheral and CNS compartments could alleviate their damaging effect on target cells (neurons, OLs) and thus promote neuroprotection. As for the possible interaction between NGF and S1P (NGF stimulates Sphk1 activity via TrkA receptors and increases intracellular S1P [162,163]), the observed increased NGF plasma levels are apparently not sufficient to promote enough synthesis of S1P in order to compensate the lowering effect of TPE on S1P plasma levels.

Hence, the clinical rationale for TPE is that there is a permeable BBB in acute demyelinating attacks, and the pathogenic substances can pass through it in both directions. The removal of plasma antibodies and immune complexes by TPE may facilitate their efflux and clearance from the CNS compartment, especially in MS patients with a highly active disease (involving the progressive forms of MS) [164]. The purpose of a relapse treatment is to accelerate functional recovery after inflammatory demyelination, alleviate the severity of the relapse, and decrease the development of persistent neurologic deficit [165]. If patients are unresponsive to initial corticosteroid treatment, which occurs in 20–25% of all cases, after an interval of 10–14 days a second corticosteroid pulse therapy in combination with TPE is recommended. TPE in steroid-refractory acute attacks/relapses is recommended as an adjunctive treatment by the American Academy of Neurology (AAN) (Level B recommendation) [166] and as a second-line treatment by the American Society for Apheresis (ASFA) (Category II; Grade 1B: strong recommendation, moderate quality evidence) [148]. In this acute clinical setting, a course of 5–7 TPE procedures over two weeks has a response rate of more than 50% [141]. In contrast to the ASFA recommendations, the AAN evidence-based guideline does not recommend TPE for chronic PP or SP forms of MS (Level A recommendation) [166]. It is noteworthy that a recent retrospective study revealed a 50% response rate for the PP/SP subgroup of patients with MS, treated with TPE/IA (both IA and TPE were equally effective) [167]. This observation implies that TPE could also be considered as escalation therapy in progressive MS [165]. In addition, another current retrospective study suggests that the escalation towards TPE should be as early as possible. It is important to point out that the delay between the onset of relapse and the initiation of TPE is crucial for the clinical response to TPE. A 7-day delay was reported to reduce the probability of TPE response by more than 30%. A delay of 14 or 21 days (routine clinical practice) results in a twofold to threefold reduction in the chance of clinically meaningful improvement [168]. All this points to a therapeutic window corresponding to the pathologically permeable BBB during and immediately after the acute demyelinating attack, which, if not used in proper time, reduces the chances of TRE for partial or complete resolution of the active MRI lesions in the great majority of treated patients [169]. This is usually accompanied by a significant improvement in the EDSS scores in post-TPE patients [170].

In addition, we carried out a second-line nanomembrane-based TPE in steroid-refractory MS in 15 patients with RR form of MS [146,147,149,171] and in one patient with progressive MS [149]. Our short-term therapeutic algorithm included 4 sessions of nanomembrane-based TPE with LVPE mode, 0.8 TPV exchange (Figure 6—our MS with LVPE, in TPE file), performed every other day, followed by 5th TPE after 1 month, 6th TPE 3 months later, and 7th TPE 6 months later [171,172]. After the application of a cycle of 4 TPE, usually the symptoms of ocular and vestibular motor function, of visual acuity, of walking without assistance, etc., as well as those of acute neurological deficit (Kurtzke’s EDSS) were improved significantly. The latest are in line with the EDSS improvements reported by other authors [170]. A significant reduction of the markers of oxidative stress (published previously) was observed as well [58].

TPE is an efficacious and safe method for the treatment of neurological disorders [58,173]. Nevertheless, its use for acute MS relapses is still modest according to recent UK clinical practice data [173]. Our nanomembrane-based experience suggests a new opportunity in technical terms and is another argument for TPE’s extended use in the field. However, it should be interpreted with caution and should be placed in the context of local specificities regarding study population, experience, availability, and insurance coverage [58,149].

Our observations on the plasma levels of immunoglobulins, NGF, and S1P present for the first time insight into the multifaceted role of TPE in the treatment of MS acute demyelinating attacks. Further research is necessary to determine their possible role as reliable biomarkers for appropriate TPE protocols.

## 8. Summary of Achieving Neuroprotection in MS

**Figure 17:**
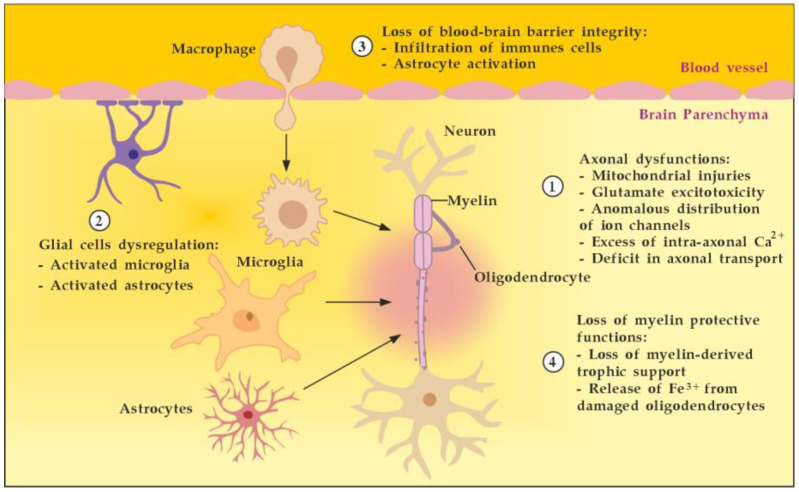
Main targets to achieve neuroprotection in MS. Adapted from [175] and modified.

In summary, achieving neuroprotection in MS is a multifaceted task requiring drugs or a combination of drugs, with different mechanisms of action, aimed at promoting axonal function (1), glial regulation (2), BBB myelin integrity (3), and restoration of myelin-protective functions (4) (Figure 17). TPE, without being considered as an alternative to the available disease-modifying drugs or new drug formulations in development, may selectively help to advance neuroprotection in all four directions. As described above, TPE could modulate the CNS microenvironment by reducing oxidative stress (less excitotoxicity), by removing the pathological antibodies and immune complexes (less OLs loss, less microglial activation, less neuronal and astrocyte apoptosis, less BBB disruption, etc.), by increasing NGF (shifting TNF-α signaling from TNFR1-mediated apoptosis to TNFR2-mediated protection and survival, steering glia toward an anti-inflammatory phenotype with less secretion of inhibitory molecules, promoting via Erk1/2-MAPK signaling pathway OLs differentiation, myelination, and remyelination, etc.), and by decreasing S1P (leading to the inability to maintain the circulatory S1P gradient and to accumulation of mature T cells in lymphoid organs, decreasing the expression of pro-inflammatory cytokines in inflammatory sites, minimizing S1P promotion of presynaptic calcium overload and cell death, etc.) [54,55,56,57,58,71,108,160,174].

## 9. Limitations and Future Directions

The main drawback of TPE is that this is an unspecific blood purification technique for removing plasma without special processing for removing only pathological factors and then replacing the separated plasma with fluids. As a result, some beneficial components, such as antibodies or cytokines with remyelinating features, are eliminated during the procedure as well [10]. Another drawback is that direct evidence for the pathogenic role of serum antibodies in MS is complicated by the marked heterogeneity of the disease and the variability of experimental procedures [7]. In addition, lessons learned from failed phase II–III trials of antibody therapies in MS that were discontinued for various reasons or withdrawn from the market taught us that there is a risk that agents that show promise in preclinical work may not translate into beneficial effects in humans [8,176]. A well-known approach from real clinical practice is to look for evidence of pathogenicity through the effect of the treatment administered. Following best practices in the field, lowering antibody levels alleviates the disease. Therapies that reduce inflammation and the immune response relieve MS symptoms and treat relapses, including prednisone, methylprednisone, apheresis, and ocrelizumab [177]. Regardless of the considered limitations, during acute demyelinating attacks with fulminant lesions and a predominant pro-inflammatory response, the positive effect of TPE administration [7] far outweighs the disadvantages of this therapeutic approach.

At present, we can search for evidence of the effectiveness of TPE based on the relief of MS symptoms from the reduction of pathological substances [177] or after evaluating the percentage of patients who achieved confirmed improvement in disability using the Kurtzke EDSS [176]. From a personalized medicine perspective, a new step in evaluating the effectiveness of new TPE technologies (including the nanomembrane-based one in particular) could be through the use of markers of axonal damage such as the level of neurofilaments in the serum before and after apheresis for acute demyelinating MS attacks [178]. Further research is needed to determine which patients benefit most from this advanced TPE treatment.

## 10. Conclusions

In conclusion, given the accelerated discovery of novel characteristic autoantibodies, in the near future, it would be expected to see an increase in the number of clinical TPE applications in the field [179,180]. Altered plasma levels of the reviewed molecular compounds in response to TPE treatment of acute MS attacks reflect a successful reduction of the pro-inflammatory burden at the expense of an increase in anti-inflammatory potential in the circulatory and CNS compartments. Plasmapheresis for MS in the twenty-first century should be taken by MS patients.

## Figures and Tables

**Table 1:** Expression of NGF and its receptors in the human immune system.

	Cellular Source	Target Receptor	Target Cell
β-NGF (mature)	mast cells, monocytes, macrophages, eosinophils, granulocytes, basophiles,	TrkA	T cells, macrophages
	T cells, B cells	p75NTR and TrkA	B cells, mast cells

**Table 2:** NGF mechanisms of action during CNS inflammation and injury.

Target	Effect
Immune system	Modulation of immune system via enhanced sympathetic innervation of lymph nodes with indirect effect decreasing CD4+ and CD8+ proliferation
BBB	Maintenance of BBB integrity
Lymphocytes	Switch to the anti-inflammatory phenotype by avoiding cytotoxicity and inducing immunosuppressive cytokines (IL-10, TGF-β)
Macrophages/ microglia	Decrease in antigen presentation by macrophage/microglia by reducing the expression of major histocompatibility complex (MHC) molecules; Shift from pro-inflammatory M1 to anti-inflammatory M2 phenotype
Astrocytes	Inactivation of toxic astrocytes mediators; Attenuation of astrogliosis, shift from pro-inflammatory A1 to anti-inflammatory A2 phenotype
OLs	Promotion of proliferation, migration, maturation, and survival of OPCs
Neurons	Promotion of axonal survival during inflammation; Upregulation of axonal LINGO1 with inhibition of axonal receptivity to oligodendrocyte myelination

## Data Availability

All data are available under request to corresponding author Dimitar Tonev (dgtsofia@abv.bg).
